# Reversible Microfluidic Platform for Spheroid Culturing, Downstream Characterization, and Dynamic Anticancer Susceptibility Testing

**DOI:** 10.1021/acsmeasuresciau.6c00140

**Published:** 2026-07-10

**Authors:** Iris Renata Sousa Ribeiro, Pedro Henrique Nunes da Silva, Bruna Gabrielle Olsen, Katarina da Silva Vilarins, Eloisa Maria Amador Souza, Isabela Aparecida de Araujo Andreotti, Leandro Ramos Souza Barbosa, Emanuel Carrilho, Angelo Luiz Gobbi, Diego Stefani Teodoro Martinez, Renato Sousa Lima

**Affiliations:** † Laboratório Nacional de Nanotecnologia, 215006 Centro Nacional de Pesquisa em Energia e Materiais, Campinas, São Paulo 13083-970, Brazil; ‡ Universidade de São Paulo, Instituto de Química de São Carlos, São Carlos 13566-590, Brazil; § Instituto de Química, Universidade Estadual de Campinas, Campinas, São Paulo 13083-970, Brazil; ∥ ILUM − Escola de Ciências, Campinas, São Paulo 13087-548, Brazil; ⊥ Instituto de Física, 28133 Universidade de São Paulo, São Paulo 05508-090, Brazil; a Instituto Nacional de Ciência e Tecnologia de Bioanalítica - Lauro Kubota, Campinas, SP 13083-970, Brazil; ∇ Centro de Ciências Naturais e Humanas, Universidade Federal do ABC, Santo André, São Paulo 09210-580, Brazil

**Keywords:** Microfluidic platform, 3D cell spheroids, Reversible PDMS bonding, Dynamic perfusion culture, Drug susceptibility testing

## Abstract

Despite the potentialities of three-dimensional (3D) spheroids to improve the predictive capability of preclinical *in vitro* assays, their translation into microfluidic drug screening pipelines remains limited by insufficient operational standardization, restricted access to downstream analyses, and static exposure regimes that inadequately capture *in vivo* mass transport. To address these downsides, we here introduce a reversibly bonded polydimethylsiloxane microfluidic platform that provides highly reproducible microwell-enabled spheroid generation, nondestructive spheroid retrieval for advanced off-chip characterization, and dynamic perfusion-based drug-susceptibility testing. A standardized operational workflow addressing practical aspects of microfluidic 3D culture (i.e., bubble suppression, surface passivation, and controlled media handling) is delivered, ultimately enabling reliable, user-friendly spheroid formation into daily practice across distinct biological systems. To date, the system has been successfully interrogated for the generation of spheroids derived from breast tumor and zebrafish liver cells. Importantly, as-produced spheroids could be retrieved from the reversible microfluidic device for downstream analyses, as demonstrated by transmission electron microscopy (TEM). TEM analysis of retrieved spheroid sections revealed preserved cellular ultrastructure, including well-defined nuclei and organelles. This data highlights the suitability of the microfluidic platform for downstream high-resolution characterization of as-cultured spheroids. Finally, doxorubicin susceptibility experiments under physiologically relevant perfusion conditions revealed enhanced cytotoxicity compared with static conditions, highlighting the critical role of flow-driven mass transport in generating more predictive drug-response profiles. Collectively, this work yields a scalable, reproducible, and accessible microfluidic framework that bridges spheroid culturing, downstream characterization, and dynamic pharmacology, expanding the analytical potential of *in vitro* microphysiological models for susceptibility testing. These assays can be broadly leveraged beyond drug discovery, including applications in toxicity testing of (bio)­chemicals and (nano)­materials toward new approach methodologies, safety research, and environmental assessments.

## Graphical abstract

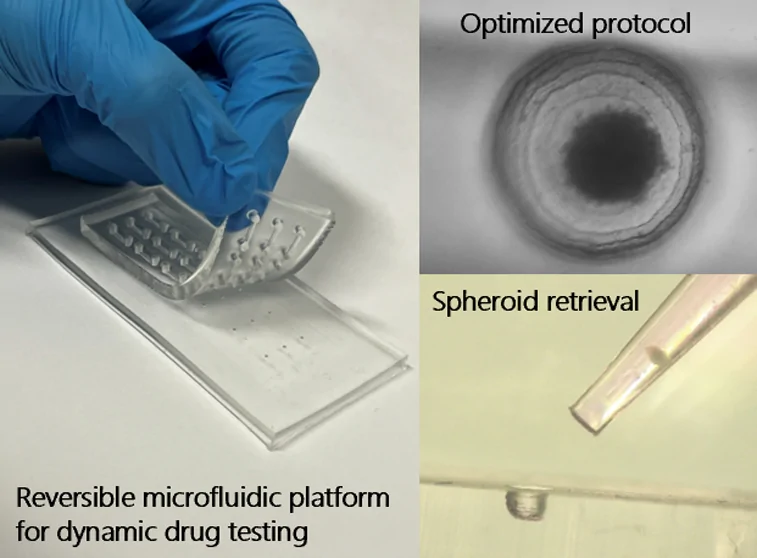

## 1 Introduction

The urgent demand for high-accuracy predictive preclinical models has driven intense efforts to move beyond traditional two-dimensional (2D) cultures and animal experimentation aiming at mitigating the high attrition rates observed across drug development processes. In oncology, e.g., the likelihood of approval (LOA) for drugs entering phase I clinical trials is only 6.7%, the lowest among all therapeutic areas, and nearly half the LOA of nononcology drugs.^1–3^ In this way, three-dimensional (3D) cell culture systems have emerged as advanced platforms for accurately studying cellular behavior, bridging the gap between conventional 2D cultures and animal models.^4^ While 2D cultures remain widely used due to their simplicity, low cost, and ease of environmental control, they fail to reproduce many critical aspects of the *in vivo* microenvironment. These cells often lose their native phenotype and polarity, compromising signaling pathways and responsiveness to external cues. Moreover, their planar architecture does not simulate *in vivo* gradients of nutrients, metabolites, gases, or signaling molecules, nor does it fully recapitulate essential cell–cell and cell–matrix interactions. As a result, the *in vitro* findings may not accurately reflect *in vivo* outcomes.^3,5–7^ Regarding animal models, although physiologically relevant, they are time-consuming, expensive, and subject to ethical concerns. Moreover, interspecies differences that can limit translational accuracy. In this sense, 3D cultures have appeared as a complementary approach, providing more physiologically relevant conditions while reducing the reliance on animal experimentation.^4,5,7–9^

Among the various types of 3D cultures, spheroids stand out as an up-and-coming alternative, demonstrating high prediction capabilities when it comes to *in vivo* angiogenesis, potency, differentiation, and survival. They are formed by the aggregation of cells (cell lines or primary cultures) in a manner that promotes their self-assembly into a spherical configuration, closely mimicking the microenvironment of native tissues.^7,10,11^ This property makes spheroids highly valuable for a range of applications, spanning from anticancer drug development and precision oncology to studies using nontumoral cells for health and environmental researches.^9–11^ Conventional approaches for spheroid formation include magnetic bioprinting, cell aggregation using Cellbed, U-bottom plates, the hanging drop method, and low-attachment tissue culture flasks.^3,12^ While valuable, these methods often incur a broad distribution of spheroid diameters, require laborious handling procedures, offer low throughput, and rely on static environments that rapidly deplete oxygen and nutrients, undermining the prediction accuracy of *in vitro* model-based analyses and ultimately underscoring the need for more standardized and dynamic culture systems.^12,13^

Microfluidic devices bring crucial advances for 3D culture-based *in vitro* screening. To begin with, they enable rapid, cost-effective, and precise production of spheroids while offering a fine control of the microenvironment.^5,12,14^ The ability of microfluidics to introduce controlled flow regimes is of pivotal relevance by improving mass transport of oxygen, nutrients, metabolites, and chemotherapeutic agents, therefore generating more physiologically relevant conditions.^15,16^ Such dynamic environments enhance the accuracy of *in vitro* pharmacological assays in predicting *in vivo* responses. Microfluidic platforms are also compatible with automation systems, streamlining protocol standardization, reducing operational complexity, and enabling parallelized testing.^15–18^ In addition, the possibility of integrating multiple interconnected microfluidic modules within a single device represents a significant advantage by enabling multiplexed susceptibility assays under flow.^13,19,20^

Among the materials used in microfluidic device fabrication, polydimethylsiloxane (PDMS) stands out due to its biocompatibility, transparency, low cost, and fast prototyping.^21^ Spheroid formation employing microfluidic PDMS devices can be performed via diverse strategies that support aggregation and maintenance of 3D structures, e.g., hydrodynamic traps, microwells, droplet generators, and micropillar arrays.^5,19,22^ In these cases, the inner PDMS surfaces are commonly coated with antiadhesive agents to prevent on-substrate cell attachment, therefore promoting cellular self-aggregation toward 3D culturing. Microwell-based approaches, in particular, offer advantages such as simple layouts, high reproducibility, and precise tuning of spheroid diameters based on the well geometry.^13,20,23,24^

Despite their advantages, most PDMS microfluidic devices face drawbacks that limit their broad applicability. First, they rely on irreversible bonding, which prevents the direct retrieval of as-produced spheroids for downstream analyses, such as on-chip staining, bulk molecular assays, and imaging.^25,26^ Conversely, while reversible bonding allows spheroid recovery by manually detaching the pieces of microfluidic devices, reported approaches often struggle to meet the trade-off between ease of disassembly, bonding strength, and reliable operation under flow conditions.^25,27,28^ Second, despite the availability of spheroid-based microfluidic platforms, comprehensive and standardized protocols to guide 3D culturing remain scattered throughout the literature, hindering their broader adoption. Critical spheroid formation-related aspects include bubble management, media exchange, well design, cellular adhesion control, and integration with parallelized culture devices.^5,29^ Finally, it is also worthwhile to underline that the literature usually fails to demonstrate susceptibility tests under dynamic conditions, a crucial attribute to ensure physiologically relevant assays, as described before.^15,16^

Addressing the previously mentioned obstacles would noticeably contribute to the widespread use of microwell-based microfluidic PDMS devices toward 3D culturing in daily practice. In this regard, we here present a set of contributions to facilitate the daily implementation of microwell-based microfluidic 3D culturing platforms, i.e., (i) the development of a standardized, user-friendly, and reproducible protocol for culturing different types of spheroids; (ii) the employment of a reversible bonding strategy to enable spheroid retrieval and downstream characterization; and (iii) the accomplishment of susceptibility tests under dynamic perfusion using a flow system. Together, these advances provide an accessible and standardized framework for integrating microfluidic 3D culturing into pharmacological and toxicological workflows in academia and industry.

More specifically, we designed a reversibly bonded microfluidic PDMS device integrating microwells, which offer precise control over spheroid geometry. The devices were prepared by cost-effective replica molding fabrication using 3D-printed masters. We established a unified set of operational protocols to address crucial steps, i.e., device fabrication, bubble management during medium addition, medium exchange volumes, prevention of undesired cell adhesion to ensure spheroid formation, and flow analysis routine. These processes were assessed to boost reproducibility and cross-laboratory accessibility, as they represent key barriers to the widespread adoption of microfluidic 3D culture platforms.

When it comes to reversible bonding, it was achieved after simple manual attachment of PDMS pieces, with adhesion strength resulting from hydrophobic interactions. No fluid leakage was observed due to the low flow rates required for the cell assays. This bonding approach allowed us to retrieve spheroids for posterior characterization in a user-friendly way—with the aid of a micropipette, thus expanding the range of downstream analyses beyond what is feasible in irreversibly bonded devices. In practice, breast tumor spheroids were collected and then imaged by transmission electron microscopy (TEM). The ability to retrieve intact spheroids is particularly valuable for investigating subcellular alterations and is often limited in irreversibly bonded microfluidic systems. TEM imaging of retrieved spheroid sections confirmed the preservation of key intracellular structures, including nuclei and organelles, demonstrating the applicability of the platform to downstream high-resolution characterization.

The device could support both micropipette-aided operations and perfusion-based assays, which revealed negative deviations in cell viability when compared with stationary-regime susceptibility testing of breast tumor spheroids after exposure to doxorubicin (DOX). This outcome makes clear the importance of conducting these experiments under a more physiologically relevant environment (i.e., flow-driven mass transport) by boosting the accuracy to predict *in vivo* response profiles. Apart from the formation of breast tumor spheroids, one should also note that the device was successfully interrogated with the generation of zebrafish liver cells (ZFL), which is an encouraging indicator of the broad applicability of the developed platform. To date, susceptibility testing of ZFL spheroids can be used across (nano)­toxicology, (bio)­chemical safety, and environmental assessments.^11,30–34^

## 2 Results and Discussion

### 2.1 Reversible Microfluidic Devices

Reversible PDMS microfluidic devices consisted of two complementary pieces that originated 18 wells interconnected by rectangular channels (Figure 1A). The final dimensions of pieces were 9.5 × 4.5 cm and 7.5 × 2.5 cm, respectively. The wells exhibited a diameter of 840 μm, while the microchannels displayed a width of 940 μm (Figure 1B). After curing and demolding the PDMS pieces, they were reversibly bonded, ensuring the device’s structural integrity and enabling subsequent spheroid characterization under controlled conditions.

**Figure 1:**
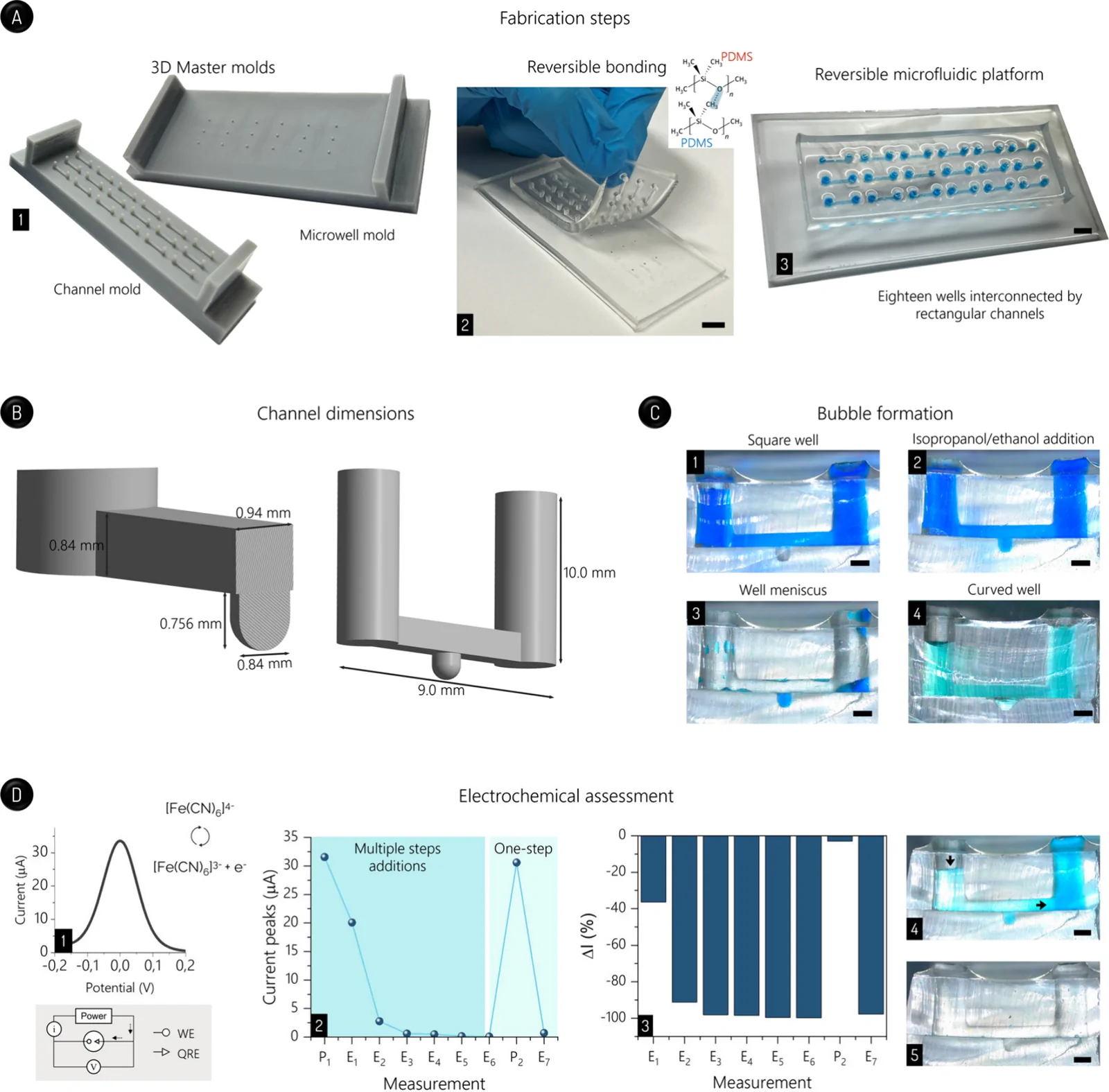
**Reversible microfluidic chip design, conditioning strategy, and electrochemical setup for medium exchange evaluation.** (**A**) Reversible microfluidic chip. 3D master molds (1), bonded device (2), and final reversible PDMS microfluidic device comprising 18 interconnected wells (3). Scale bars: 1 cm (2) and 5 mm (3). Inset: chemical structures depicting the hydrophobic PDMS–PDMS interactions enabling reversible sealing. (**B**) Schematics of the chip layout highlighting the square culture wells (side length: 0.84 mm) and the interconnecting rectangular microchannels (width: 0.94 mm). (**C**) Chip conditioning strategy for well filling. Incomplete filling with air bubble entrapment during direct aqueous medium loading (1), complete filling after ethanol or isopropanol preconditioning (2), filled well with liquid retained after removal of liquid from the microchannel (3), and an alternative curved-well design evaluated to reduce bubble formation (4). Scale bars: 1 mm. (**D**) Electrochemical monitoring of medium exchange. SWV scan of the [Fe­(CN)_6_]^3–^/^4–^ redox probe (5 mmol L^–1^) using a potential window from – 0.2 to +0.2 V (1), voltammograms acquired after successive exchange steps (E1–E6) following an initial probe-filled condition (P1), and after a single-step addition of 125 μL (P2/E7) (2), corresponding peak current differential from P1 and P2 (3), and optical images obtained using a dye solution immediately after dye addition and after five washing steps with water (4,5). Data represent mean ± standard deviation (SD, *n* = 3). Insets: generic circuit of the two-electrode on-MEC cells and reactions involving the probe. Scale bars: 1 mm.

The devices had sufficient adhesion to withstand the perfusion conditions used throughout this study, ensuring leakage-free assays. To mathematically assess the adhesion strength of the reversible bonding strategy, burst-pressure measurements were performed using a PDMS microfluidic channel with decreased dimensions, i.e., 140.0 μm (width), 45.0 μm (height), and 25.0 mm (length). Such a new dimension was adopted to enable the instrumental setup to detect the device-tied variations in hydraulic resistance (Supporting Information). The reversibly bonded PDMS/PDMS device remained leakage-free up to 150 μL min^–1^, corresponding to a burst pressure of 63 kPa (Figure S1).^35^ This outcome is on par with the data reported for reversibly bonded microfluidic devices.^36^

### 2.2 Procedure to Mitigate the Bubble Formation Issue

Upon introducing the aqueous medium into the channel, the first issue observed was the incomplete filling of the wells due to bubble formation (Figure 1C and Video S1). To circumvent this limitation, the device was prefilled with ethanol prior to medium addition. The effectiveness of this approach is consistent with previous reports showing that alcohols improve PDMS wettability and facilitate liquid entry into hydrophobic microstructures.^37^ In particular, ethanol exhibits a markedly lower static contact angle on untreated PDMS (∼28°) compared to water (>90°), a difference that reflects its amphiphilic interfacial behavior and enables more efficient displacement of trapped air within confined geometries.^37^ As a result, this pretreatment with ethanol proved to be efficient in mitigating bubble formation within wells when manually adding the solution with a micropipette (see Figure 1C and Video S2). Following medium loading could then be performed without undergoing bubble-related issues. It is worth noting that an alternative strategy was also tested to address this limitation, consisting of demolding more curved wells (see Figure 1C and Figure S2). Although this design successfully minimized bubble entrapment, the resulting spheroids were easily dragged out of the well during medium exchanges, compromising culture stability. Based on the above outcomes, the square-well format combined with ethanol preconditioning was selected for all subsequent experiments, as this configuration ensured complete filling of wells and spheroid retention along cellular proliferation and drug susceptibility testing.

### 2.3 Evaluating the Medium Exchange Volume

To ensure reliable operation of the microfluidic workflow, we evaluated the volume required to guarantee a complete fluid replacement within the whole channel, as incomplete medium exchanges may lead to carryover effects, hence compromising reproducibility. The fluid exchange was assessed by electrochemically monitoring electrolyte and a solution of 5 mmol L^–1^ ferri/ferrocyanide ([Fe­(CN)_6_]^3–^/^4–^) redox couple as an electroactive tracer. For these analyses, we used a meshed electrochemical chip (MEC), as recently developed by our group.^35,38–41^ MEC consists of an array of microfabricated two-electrode sensors (working electrode, WE, and quasi-reference electrode, QRE) patterned on a glass wafer. This architecture eliminates the need for external electrodes and enables reproducible measurements using microliter-scale sample volumes.

The microfluidic channel was initially filled with 5 mmol L^–1^ [Fe­(CN)_6_]^3–^/^4–^. Under this initial condition, as expected, square wave voltammetry (SWV) scans displayed a well-defined peak current typical of this reversible redox probe. Sequential medium exchange was then assessed by successive additions of 25 μL of supporting electrolyte to the channel, progressively diluting the probe. After each exchange step, the channel content was collected and analyzed by SWV. Because of the gradual [Fe­(CN)_6_]^3–^/^4–^ dilution, the current peaks decreased systematically with increasing number of exchange steps (Figure 1D). An efficient renewal of the channel content was reached after five consecutive exchange steps (total exchange volume: 125 μL), with the SWV peak became negligible, which indicates the presence only of electrolyte in the channel. This conclusion is further supported by the relative current variation relative (ΔI), which exhibited a pronounced drop during the first exchange cycles, followed by a plateau after the fifth step (see Figure 1D). The emergence of this plateau suggests that additional exchanges yield only marginal changes in electrochemical response, consistent with the channel being effectively filled with probe-free electrolyte beyond this point. In consistency, a single addition of 125-μL probe caused the voltametric sensor to practically recover the initial SWV peak.

In agreement with the electrochemical findings, optical images acquired by filling the channel with a dye solution corroborated the progressive replacement of the initially colored liquid by water, with the channel becoming visually transparent after five exchange steps (see Figure 1D and Video S3). These results establish that the total volume of 125 μL is sufficient to yield the complete renewal of the microwell-based channels.

### 2.4 Channel Coating toward Spheroid Proliferation

After conditioning the chip with ethanol, the next step was to introduce the cells to promote spheroid formation. Initial attempts revealed that the cells readily adhered to the PDMS surface, particularly along the microchannels, thereby hindering the establishment of uniform 3D cultures. To address this issue, a surface passivation study was conducted to mitigate cell adhesion. Among the strategies reported in the literature, overnight incubation with 10% bovine serum albumin (BSA) is one of the most common and effective approaches for reducing nonspecific cell attachment to PDMS surfaces.^24^ BSA adsorbs onto the hydrophobic PDMS through a combination of hydrophobic and electrostatic interactions, forming an antifouling layer that prevents the substrate from coming into direct contact with cells. However, cell adhesion still occurred at the channel inlets after overnight exposure to 10% BSA (Figure 2A). Accordingly, previous studies have demonstrated that BSA provides only transient surface blocking and is ineffective in preventing long-term on-PDMS adsorption.^42^ To overcome this limitation, Pluronic F127 was employed as an alternative antipassivation agent. Pluronic adsorbs onto hydrophobic PDMS via its central hydrophobic poly­(propylene oxide) (PPO) block, while the hydrophilic poly­(ethylene oxide) (PEO) tails form a hydrated brush that avoids protein- and cell-induced fouling.^43^ Although the literature reports multiple Pluronic F127 passivation protocols under different concentrations, incubation times, and temperatures,^43,44^ the procedure was standardized under cell culture-relevant conditions (37 °C) using 1% formulation. Overnight incubation resulted in more effective surface passivation than 5-h incubation (Figures 2A and S3), indicating that longer exposure enables more complete adsorption of Pluronic molecules, as expected.^24^ This extended adsorption led to a denser and more stable passivation layer, which is expected to enhance the resistance to protein and cell adhesion. Notably, the reduced cell adhesion observed after Pluronic F127 treatment was consistently reproduced across independent experiments (*n* = 5), reinforcing the efficacy of this passivation strategy.

**Figure 2:**
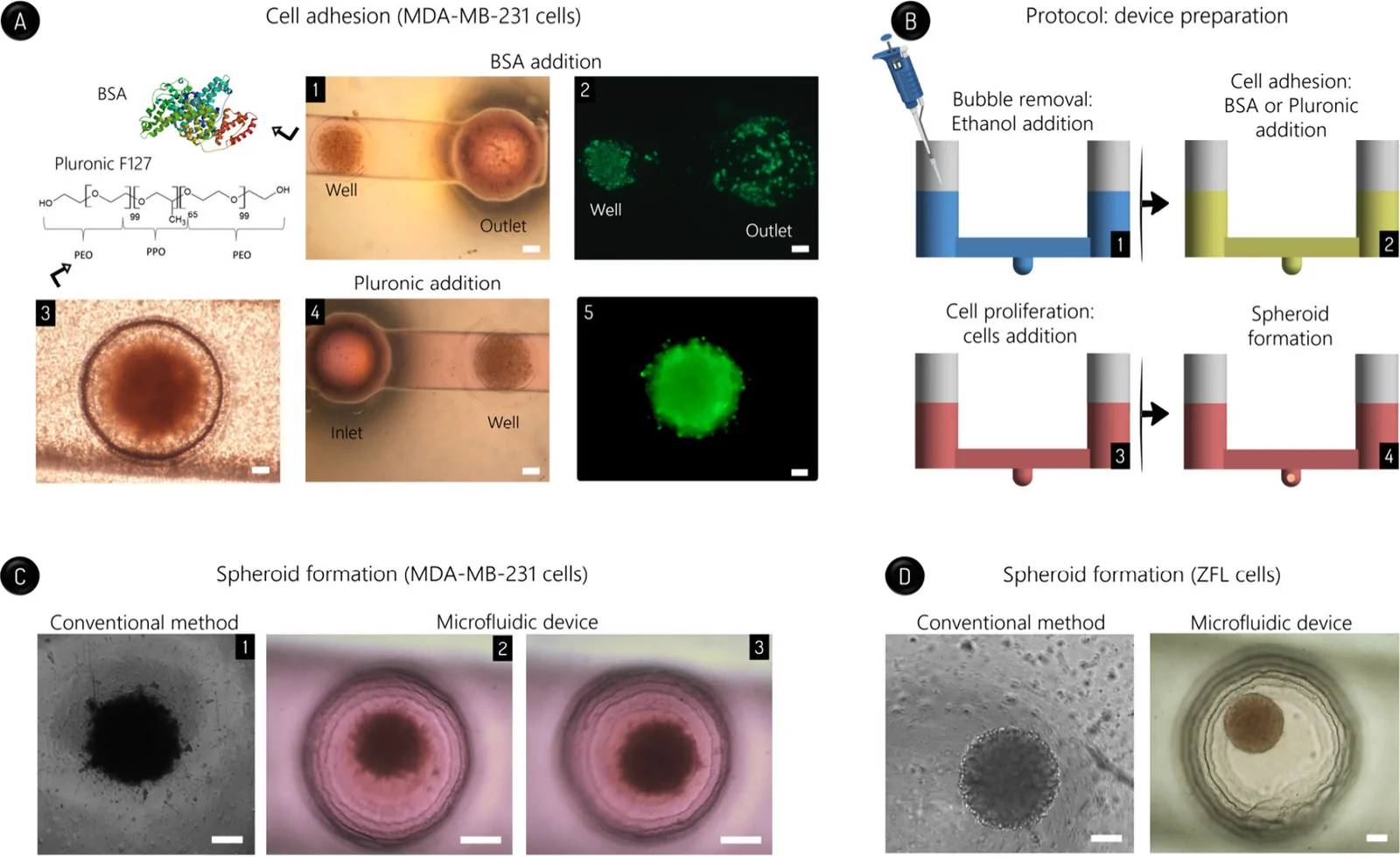
**Surface passivation and spheroid formation in the reversible microfluidic device.** (**A**) Evaluation of PDMS surface passivation strategies. Representative bright-field (1) and fluorescence (2) images showing cell adhesion of MDA-MB-231 cells at the microchannel outlet after overnight incubation with 10% (w/w) bovine serum albumin (BSA), indicating insufficient suppression of cell attachment. Representative bright-field (3,4) and fluorescence (5) images after overnight incubation with 1% (w/w) Pluronic F127 at 37 °C, showing markedly reduced cell adhesion at the channel inlet (*n* = 5). Green fluorescence corresponds to live cells (Calcein-AM staining). Insets: chemical structures of BSA and Pluronic F127. Scale bars: 300 μm (1,2,4) and 100 μm (3,5). (**B**) Device preparation protocol. Channel conditioning with ethanol (1), addition of BSA or Pluronic F127 for surface passivation (2), cell seeding (3), and spheroid formation (4). (**C**) MDA-MB-231 spheroids obtained using a conventional static culture method (1, U-bottom plate) and within the reversible microfluidic device (2,3). Scale bars: 200 μm. (**D**) Zebrafish liver cell spheroids obtained using a conventional static culture method (1) and within the reversible microfluidic device (2). Scale bars: 100 μm.

After conditioning the device with ethanol and coating the PDMS surfaces with the antipassivation agent after overnight Pluronic F127 incubation, cells were introduced into the microchannel toward spheroid formation (Figure 2B). Different cell densities were tested, with 1.0 × 10^5^ cells per channel being selected as the optimal condition since higher concentrations did not yield significant differences in spheroid density, morphology, or size (Figures 2C and S4). It is worth noting that the spheroids generated in the microfluidic device exhibited characteristics comparable to those produced using the conventional U-bottom plate-based culture method, with average diameters of 0.45 ± 0.02 mm and 0.55 ± 0.03 mm, respectively (*n* = 5 spheroids per condition). To demonstrate the platform’s versatility, the system was also challenged with the formation of zebrafish liver cells. These cells formed spheroids at 1.5 × 10^5^ cells per channel, which were also morphologically and dimensionally similar to those generated by the standard technique (Figure 2D).

Importantly, the ability of the platform to consistently produce spheroids with comparable morphology and size to conventional methods supports its reliability as a culturing approach, while enabling integration with additional functionalities inherent to microfluidic systems.

### 2.5 Downstream TEM Characterization of As-Produced Spheroids

The reversible bonding strategy enabled the gentle retrieval of spheroids from the microfluidic device, which is generally impractical in irreversibly bonded PDMS systems. More specifically, the medium in the channel was first aspirated using a micropipette, while remaining a volume within the well.^45^

Following, PDMS pieces were detached and then the cells were collected with the aid of the micropipette (Figure 3A). Macroscopic and microscopic inspection during retrieval indicates that spheroids remained cohesive, without apparent disruption or disaggregation (Figure 3A and S5). The retrieved spheroids were subsequently analyzed by high-resolution TEM. Their ultrastructural features were preserved, indicating that the collection process did not compromise cellular integrity (Figure 3B). Specifically, TEM micrographs of retrieved spheroid sections revealed well-preserved nuclei and organelles, i.e., mitochondria, lysosomes, and the Golgi complex. The presence of abundant mitochondria and organized cytoplasmic compartments indicates metabolically active cells.^46^ These findings demonstrate compatibility with downstream high-resolution analyses. One should also note that accessing retrieved spheroids is especially critical when investigating drug-induced subcellular alterations. Many cellular responses, such as mitochondrial dysfunction, autophagic activation, lysosomal remodeling, and nuclear condensation, initially manifest at the ultrastructural level.^46,47^ Therefore, the ability to retrieve aggregates provides a mechanistic evaluation that would otherwise be lost in devices requiring destructive retrieval methods.

**Figure 3:**
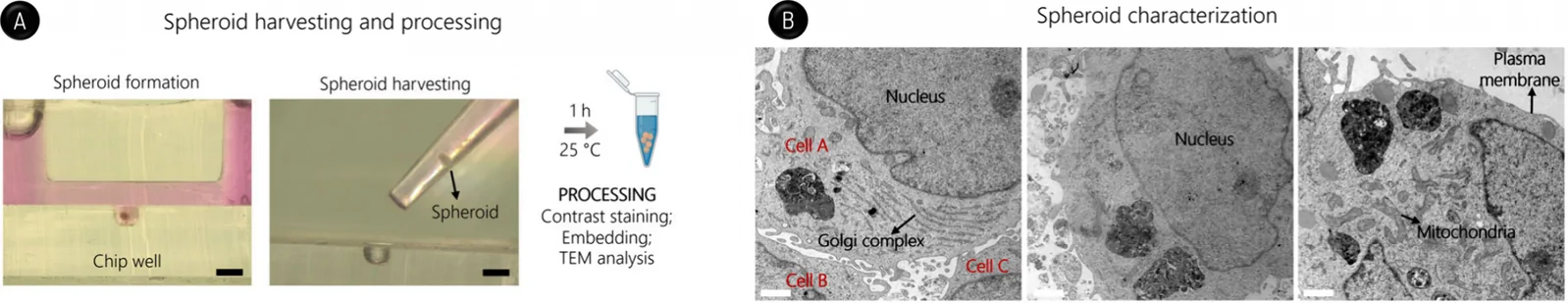
**Ultrastructural characterization of spheroids.** (**A**) TEM workflow. Schematic illustrations of spheroid retrieval and sample preparation for TEM analysis. Scale bars: 800 μm. (**B**) Spheroid characterization. Representative TEM micrographs of spheroid sections at different magnifications, including overall morphology and intracellular structures. Scale bars: 1 μm.

Together, the above-mentioned results show that the reversible microfluidic platform not only supports spheroid formation but also unlocks advanced characterization approaches, such as TEM. The integration of microfluidic culturing with high-resolution imaging expands the analytical possibilities for studying the effects of drugs, nanoparticles, and biomaterials in 3D cellular environments.

### 2.6 Drug Exposure in a U-Bottom Plate

Prior to on-chip experiments, spheroid susceptibility to DOX was systematically evaluated using a conventional static culture approach to define an appropriate concentration window and establish a robust baseline for subsequent microfluidic assays. This preliminary screening step aimed at identifying drug concentrations capable of eliciting distinct biological responses, ranging from minimal cytotoxicity to pronounced viability loss.

Based on the results, DOX concentrations ranging from 500 to 2000 nmol L^–1^ were selected for the on-chip experiments. Within this window, lower concentrations (500 nmol L^–1^) produced no detectable impact on spheroid viability, whereas concentrations higher than 1200 nmol L^–1^ induced a marked cytotoxic response. To date, the spheroid viability decreased to approximately 62% when adding 1200 nmol L^–1^ DOX, as consistently determined by both LIVE/DEAD imaging and CellTiter-Glo 3D measurements (Figure 4A). This intermediate-response regime was selected because it enables the detection of transport-dependent differences in drug efficacy, which would be masked at very low or near-lethal drug concentrations.

**Figure 4:**
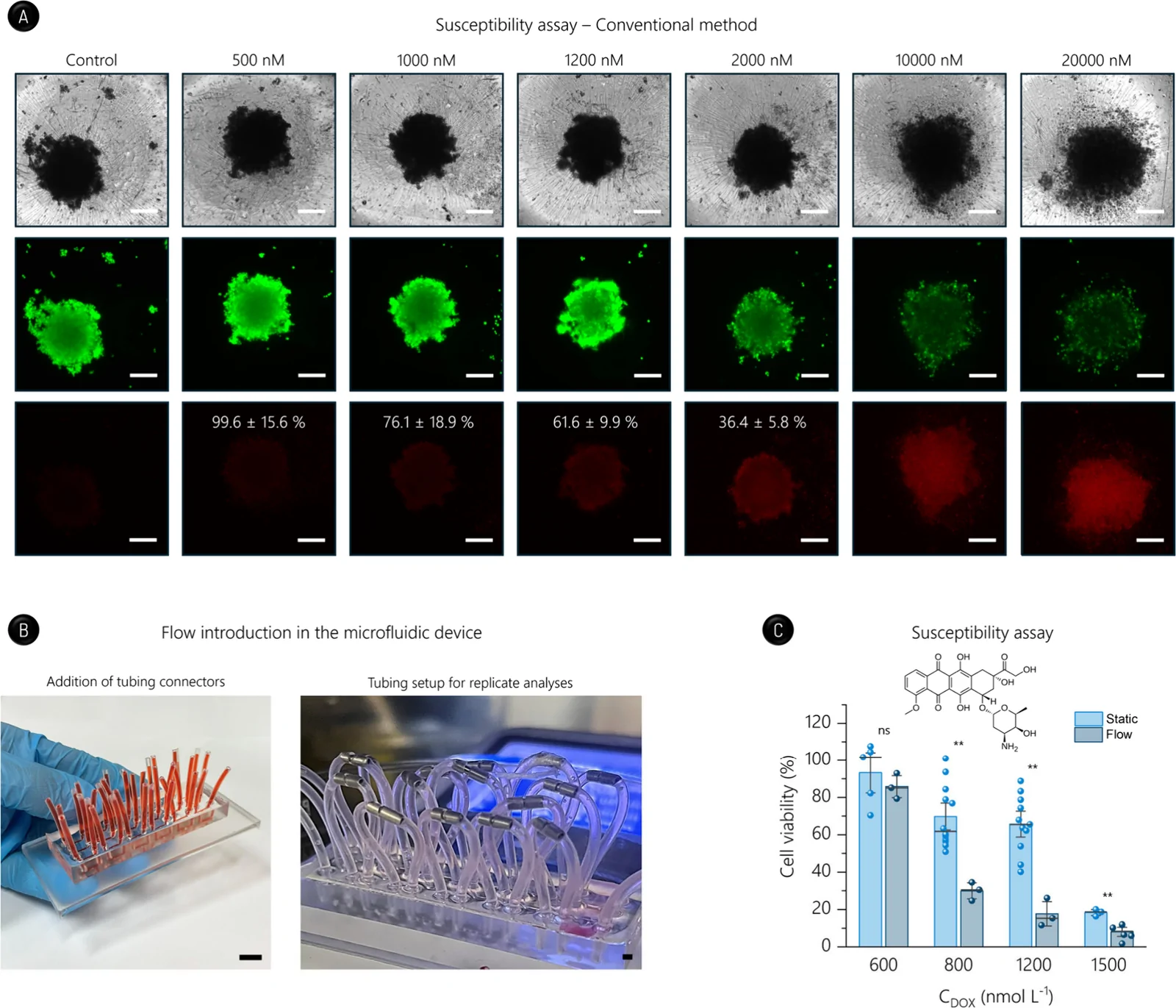
**Drug exposure under conventional and microfluidic conditions.** (**A**) Conventional drug exposure assay. Spheroid viability after treatment with selected DOX concentrations (500–20000 nM) was assessed by LIVE/DEAD fluorescence imaging (middle and bottom), where green (Calcein-AM) and red (propidium iodide) indicate live and dead cells, respectively. A representative bright-field image is shown at the top. Quantitative viability data, obtained using the CellTiter-Glo 3D assay, are presented as percentage values in the figure. Data represent mean ± SD (*n* = 6). Scale bars: 250 μm. (**B**) Introduction of dynamic perfusion in the microfluidic device. Left: addition of tubing connectors to the microfluidic chip. Right: tubing setup enabling parallel channels for replicate analyses. Scale bars: 1 cm (left) and 2 mm (right). (**C**) On-chip susceptibility assay of tumor spheroids exposed to DOX under static and perfused (flow) conditions. Spheroid viability was quantitatively assessed using the CellTiter-Glo 3D assay. Results are presented as mean ± SD, with individual data points shown in all graphical representations, and sample sizes ranging from *n* = 3 to *n* = 12 per group. Inset: chemical structures of DOX.

### 2.7 Drug Exposure in the Microfluidic Platform under Flow

Building on the dose window established under conventional conditions, we then delved into performing drug susceptibility tests in our microfluidic chips under dynamic conditions (Figure 4B and S6). Dynamic perfusion was applied at a flow rate of 2.6 μL min^–1^, corresponding to reported interstitial fluid velocities in solid tumors.^4,48^ The wall shear stress was estimated to be approximately 3 × 10^–3^ dyn cm^–2^, a magnitude consistent with the lower range of tumor interstitial mechanical stimuli.^49^ Under this condition, spheroids could be exposed to physiologically relevant fluid-induced cues without experiencing excessive mechanical stress. For reference purposes, static on-chip experiments were also performed to devise direct comparison with the prior perfused assays (Figure 4C). The viability obtained under static on-chip conditions was consistent with that previously observed under conventional culture conditions (see Figure 4A). This finding further supports the equivalence of the microfluidic system to the conventional method, not only in terms of spheroid morphology, as aforesaid, but also regarding the biological response (under static regime). Under continuous perfusion, nonetheless, statistical analysis confirmed a significantly greater reduction in spheroid viability compared to static conditions (two-tailed Mann–Whitney U test, Holm–Bonferroni correction, adjusted *p* < 0.05; see Figure 4C). This enhanced cytotoxic response is attributable to the flow-triggered shifts in mass-transport dynamics.^50^ Drug movement in static cultures is driven by diffusion, incurring the formation of local concentration gradients along the channel that can significantly hinder the penetration of DOX into the spheroid core.^4^ As a result, peripheral drug accumulation is attenuated, which may be detrimental to the efficacy of clinical treatments. In contrast, perfusion disrupts gradient formation, minimizes diffusive resistance, and continuously replenishes the drug around the spheroid interface. These features lead to a deeper drug penetration across the 3D structure, thereby explaining the more pronounced drop in viability when compared to the static assays.^50^

Consistent with previous studies,^5,14,51,52^ these findings make clear the pivotal relevance of reproducing the *in vivo*-like mass transport phenomena, e.g., controlled flow fields, physiologically relevant shear, and drug/nutrient delivery, toward more accurate predictions of *in vivo* responses. By mitigating artifacts inherent to static assays and providing more reliable drug-response readouts, flow-enabled microfluidic platforms enhance the translational relevance and may ultimately reduce the reliance on animal testing.

## 3 Conclusions

We present a versatile and accessible reversible microfluidic platform that addresses persistent limitations of current 3D cell culture and organotypic drug-screening technologies. By combining a microwell-based architecture with a reversible PDMS-bonding strategy, the system enables reproducible spheroid formation while possessing the ability to retrieve aggregates, which is of paramount significance for advanced downstream analyses of drug-induced subcellular alterations. In addition, the system allows dynamic drug susceptibility testing, hence boosting the accuracy in predicting *in vivo* responses. Beyond the device itself, this work establishes a standardized and practical operational framework for spheroid-based microfluidic assays, facilitating their broader adoption. Future efforts will be addressed to improve throughput by markedly increasing the number of microwells per device toward scaling and parallelization strategies.^13,20^ Additionally, the evaluation of a broader panel of compounds, together with a more comprehensive biological characterization of the spheroids (e.g., spatial heterogeneity and structural gradients), will be essential to further validate the platform’s applicability across diverse pharmacological scenarios.

Collectively, this platform provides a practical route for integrating spheroid-based 3D cultures into pharmacological and toxicological pipelines. By lowering technical barriers, enabling direct spheroid retrieval, and supporting dynamic exposure regimes, the platform bridges a critical gap between conventional *in vitro* models and *in vivo* studies toward new approach methodologies. As such, it holds strong potential to improve the predictive power of preclinical drug screening, reduce reliance on animal models, and accelerate the development of more effective anticancer therapies.

## 4 Material and Methods

### 4.1 Material

All the reagents were of analytical grade. Cell culture medium (DMEM) and trypsin were obtained from Vitrocell Embriolife. Fetal bovine serum (FBS; Gibco, Thermo Fisher Scientific) and penicillin–streptomycin (Gibco, Thermo Fisher Scientific) were used as received. The LIVE/DEAD Viability/Cytotoxicity Kit was also purchased from Thermo Fisher Scientific. Ultralow attachment surface spheroid microplates (Corning), phosphate-buffered saline (PBS, 10 mmol L^–1^, pH 7.4), phosphate buffer (PB), BSA, and Pluronic F127 were obtained from Sigma-Aldrich. The CellTiter-Glo 3D Cell Viability Assay was purchased from Promega. Dow Corning supplied the PDMS base and the Sylgard 184 curing agent.

### 4.2 Fabrication Process

The channel pattern in PDMS was obtained via replica molding by mixing a silicone elastomer base solution with its curing agent (Sylgard 184) at a 10:1 (w/w) ratio. Two PDMS layers were fabricated: one containing 18 microfluidic channels and another comprising 18 microwell structures. Each layer was molded by casting the PDMS mixture onto 3D-printed master molds (Formlab printer), which were delimited with adhesive tape, and cured at 65 °C for 90 min. After curing, the PDMS replicas were peeled off and aligned manually. The device was assembled by reversibly sealing the channel layer to the microwell layer, enabling easy assembly and disassembly as needed.

### 4.3 Adhesion Strength

A pressure controller (OB1MK4, Elveflow), a 12-channel microfluidic valve (MUX Distribution 12, Elveflow), and a mass flow meter (mini CORI-FLOW, Bronkhorst) were used to inject water at room temperature into the PDMS channels under pressure ramps while accurately monitoring the flow rates. Further details regarding the experimental setup and data analysis are provided in the Supporting Information.

### 4.4 Procedure to Mitigate the Bubble Formation Issue

Before cell loading, the assembled PDMS chips underwent conditioning to ensure sterilization and prevent bubble formation. Initially, the devices were exposed to UV light for 1 h, followed by filling all microchannels with ethanol to enhance surface wetting and eliminate trapped air bubbles.

### 4.5 Evaluating the Medium Exchange Volume

Electrochemical measurements were performed using a hand-held single-channel potentiostat (Sensit BT, PalmSens). The supporting electrolyte consisted of 1.0 mol L^–1^ KNO_3_ in 12.0 mmol L^–1^ phosphate buffer (PB). SWV measurements of the [Fe­(CN)_6_]^3–^/^4–^ redox couple (5 mmol L^–1^) were carried out using a potential window from – 0.3 to +0.3 V, a modulation amplitude of 35.0 mV, a step potential of 3.5 mV, and a frequency of 15 Hz. All analyses were performed using 0.8 mm working electrodes (WEs) integrated into two-electrode on-MEC sensors (meshed electrochemical chips, MEC).

To determine the number of medium-exchange cycles required to fully replace the liquid inside the microfluidic channel, a sequential rinsing assay was conducted. First, the channel was filled with the probe solution. At each step, 25 μL of the channel content were carefully withdrawn and deposited onto the electrode for SWV analysis, followed by the addition of 25 μL of fresh supporting electrolyte. This withdrawal/addition cycle was repeated, progressively diluting the probe solution within the channel. Replicate numbers are reported in the figure captions.

### 4.6 Channel Coating toward Spheroid Proliferation

After chip conditioning, the channels were rinsed with BSA (10% w/v in PBS) or Pluronic F-127 (1% w/v in PBS) overnight at 37 °C to minimize nonspecific cell attachment. The solution volume used was based on that applied in the electrochemical analyses. Subsequently, MDA-MB-231 (human breast tumor) and ZFL cells were introduced into the device. The number of seeded cells was adjusted for each cell line to obtain homogeneous spheroids. For tumor cells, the cell density ranged from 8 × 10^4^ to 20 × 10^4^ cells per channel, whereas for ZFL cells it varied from 10 × 10^4^ to 20 × 10^4^ cells per channel.

Spheroid culture was then maintained at 37 °C under a CO_2_ atmosphere (Forma Series 3 Water Jacketed, Thermo Scientific) for 3 days for MDA-MB-231 cells and 5 days for ZFL cells, using DMEM supplemented with 10% FBS and 1% penicillin–streptomycin solution. These incubation periods are well established in the literature for conventional spheroid formation methods.^53–55^ In practice, suspensions of either MDA-MB-231 or ZFL cells were gently introduced into the PDMS channels using a pipet. To minimize evaporation and reduce contamination risk, the microfluidic device was placed inside a larger closed Petri dish containing two smaller PBS-filled dishes to maintain a humidified microenvironment.

In parallel, spheroid formation was also carried out under conventional conditions using U-bottom, low-attachment plates for comparison purposes. To confirm cell viability and the successful formation of viable spheroids within the microfluidic device, a fluorescence-based LIVE/DEAD assay using Calcein-AM and propidium iodide (PI) was performed. Briefly, a staining solution was prepared by diluting 2.5 μL of Calcein-AM and 10 μL of PI in 5 mL of PBS. Then, 200 μL of the staining solution was added to each well, and the plate was incubated at room temperature in the dark for 20 min. After incubation, fluorescence images were acquired for analysis.

### 4.7 Downstream TEM Characterization of As-Produced Spheroids

The TEM preparation protocol used here follows procedures previously described in the literature^56^ with minor adaptations for spheroid handling. Spheroid samples were washed in PBS and fixed using Karnovsky’s fixative solution (2.5% glutaraldehyde and 4% paraformaldehyde in 0.1 M sodium cacodylate buffer) for 1 h at room temperature. After fixation, approximately ten spheroids were collected from the wells and transferred to a microcentrifuge tube (Eppendorf). The spheroids were then washed three times with 0.1 M cacodylate buffer. For TEM, the fixed spheroids were postfixed in 1% osmium tetroxide for 1 h in the dark, then washed with cacodylate buffer. Samples were dehydrated in a graded acetone series (30%, 50%, 70%, 90%, and 100%) and then infiltrated with increasing concentrations of Poly/Bed 812 epoxy resin. Complete infiltration was achieved using 100% resin, and the samples were polymerized at 60 °C for 72 h. Polymerized blocks were trimmed, and ultrathin sections (60–90 nm) were obtained using an ultramicrotome. The sections were stained with 2% uranyl acetate for 30 min, followed by lead citrate for 2 min. Ultrathin sections were examined using a JEOL JEM-1400 Plus transmission electron microscope operating at 80 kV.

### 4.8 Drug Exposure

After spheroid formation, DOX was added directly to the microwells using a pipet. Drug exposure steps were conducted in an incubator at 37 °C under a CO_2_ atmosphere for 24 h. Cell viability was assessed using the CellTiter-Glo 3D and LIVE/DEAD assays. For the CellTiter-Glo 3D assay, spheroids were washed with PBS and incubated with a 1:1 mixture of culture medium and luminescence-based viability reagent. Plates were kept at room temperature for 30 min, with gentle shaking during the first 5 min to ensure complete cell lysis. The supernatants were collected, transferred to a white 96-well plate, and luminescence was recorded. The same procedure was performed using spheroids conventionally generated in U-bottom, low-attachment plates for comparison. Replicate numbers are reported in the figure captions.

### 4.9 Flow Control

To enable fluid perfusion, tubing connectors were positioned in the master mold described in Section 4.2 prior to PDMS curing, allowing the ports to be fully integrated into the device after assembly. After bonding the PDMS layers, the device was connected to external tubing and operated using a peristaltic pump (Masterflex Ismatec 78001–82). Perfusion conditions were defined to reproduce physiologically relevant interstitial flow. Tumor interstitial flow velocities reported in the literature typically range from 3 to 55 μm s^–1^, with values approaching 100 μm s^–1^ in regions of elevated interstitial fluid pressure.^4,48^ Based on these reports, an upper-limit velocity of 55 μm s^–1^ was selected for device operation.

The corresponding volumetric flow rate (Q) was calculated as

Q=v×A

where *v* is the mean interstitial flow velocity, and *A* is the microchannel cross-sectional area (channel width *w* = 0.94 mm; channel height *h* = 0.84 mm).

$$Q=55\times 10^{-6}ms^{-1}\times 7.896\times 10^{-7}m^{2}=4.34\times 10^{-11}m^{3}s^{-1}$$

Converting this value to μL min^–1^ yields

$$Q=0.0434\mu Ls^{-1}=2.60\mu L\min^{-1}$$

Thus, operating the channel at a physiologically relevant velocity of 55 μm s^–1^ required a flow rate of approximately 2.6 μL min^–1^, which is compatible with the minimum controllable rate of the peristaltic pump used (≈2.43 μL min^–1^).

Because shear stress represents the primary mechanical cue sensed by cells under perfusion,^49^ the wall shear stress (τ) generated under these conditions was estimated. For laminar flow in rectangular microchannels:

$$\tau =\frac{6\mu Q}{wh^{2}}$$

Using a medium viscosity of approximately 0.8 mPa s (37 °C),^57^ the resulting wall shear stress was approximately 3.1 × 10^–4^ Pa (0.003 dyn cm^–2^). This magnitude lies within the lower range reported for tumor interstitial shear stress,^48^ supporting the physiological relevance of the applied perfusion conditions while avoiding excessive mechanical loading of the spheroids.

During experiments, fluids were either added directly to the microwells or perfused through the channels at this controlled flow rate. Drug exposure and viability assays were performed as described in Section 4.8; however, under perfusion conditions, DOX-containing medium was delivered through the microchannels in a recirculating flow configuration at 2.6 μL min^–1^.

### 4.10 Statistics

Nonparametric tests were applied to evaluate differences between conditions. Static and flow groups at each concentration (600, 800, 1200, and 1500 nmol L^–1^) were compared using the Mann–Whitney U test (two-tailed) for independent samples. To account for multiple comparisons, p-values were adjusted using the Holm–Bonferroni method, with statistical significance defined as adjusted p-values <0.05. Results are presented as mean ± standard deviation (SD), with individual data points shown in all graphical representations, and sample sizes ranging from *n* = 3 to *n* = 12 per group. Statistical significance levels were indicated as follows: *p < 0.05, **p < 0.01, and ***p < 0.001; ns, not significant.

## Supplementary Material









## Notes

Adhesion strength and operational limits of the reversible sealing strategy; well geometry; surface passivation using Pluronic F127; spheroid formation using different cell seeding densities; recovery of spheroids from the microfluidic channel after sequential collection steps; implementation of dynamic perfusion in the microfluidic platform using a peristaltic pump (PDF)
Video demonstration of bubble formation during aqueous medium loading (MP4)
Video demonstration of ethanol-assisted bubble removal (MP4)
Video demonstration of progressive fluid exchange within the microfluidic device (MP4)
