# Supplementary material for: Reversible Microfluidic Platform for Spheroid Culturing, Downstream Characterization, and Dynamic Anticancer Susceptibility Testing
Source: ACS Meas Sci Au. 2026 Jul 10;6(4):1339–49. doi: 10.1021/acsmeasuresciau.6c00140 (PMC13495713; doi:10.1021/acsmeasuresciau.6c00140)
Supplement: Supplementary file 1 [file tg6c00140_si_001.pdf]

Supporting Information

# Reversible Microfluidic Platform for Spheroid Culturing, Downstream Characterization, and Dynamic Anticancer Susceptibility Testing

Iris Renata Sousa Ribeiro,[1,ǂ,*] Pedro Henrique Nunes da Silva,[1,2,3,ǂ] Bruna Gabrielle Olsen,[1,3] Katarina da Silva Vilarins,[4] Eloisa Maria Amador Souza,[4] Isabela Aparecida de Araujo Andreotti,[1,2] Leandro Ramos Souza Barbosa,[5] Emanuel Carrilho,[2,6] Angelo Luiz Gobbi,[1] Diego Stefani Teodoro Martinez,[1] Renato Sousa Lima.[1,2,3,7]

[1]Laboratório Nacional de Nanotecnologia, Centro Nacional de Pesquisa em Energia e Materiais, Campinas, São Paulo 13083-970, Brazil

[2]Universidade de São Paulo, Instituto de Química de São Carlos, São Carlos 13566-590, Brazil

[3]Instituto de Química, Universidade Estadual de Campinas, Campinas, São Paulo 13083-970, Brazil

[4]ILUM – Escola de Ciências, Campinas, São Paulo 13087-548, Brazil

[5]Instituto de Física, Universidade de São Paulo, São Paulo 05508-090, Brazil

[6]Instituto Nacional de Ciência e Tecnologia de Bioanalítica - Lauro Kubota, Campinas, SP 13083-970, Brazil

[7]Centro de Ciências Naturais e Humanas, Universidade Federal do ABC, Santo André, São Paulo, 09210-580, Brazil

ǂThese authors contributed equally to this work.

*Corresponding author: iris.ribeiro@lnnano.cnpem.br

**Contents:**

1. **Adhesion Strength**
2. **Curved-well geometry**
3. **Surface Passivation (Pluronic F127)**
4. **Spheroid Formation Using Different Cell Seeding Densities**
5. **Recovery of spheroids from the microfluidic channel after sequential collection steps**
6. **Implementation of dynamic perfusion in the microfluidic platform using a peristaltic pump**
7. **Videos**

## 1. Adhesion Strength

To determine the burst pressure supported by the reversibly bonded polydimethylsiloxane (PDMS) microchannels, pressure ramps were generated by progressively increasing the flow rate (Q). Linear fitting of the pressure–flow relationship provided the hydraulic resistance (Hr), which was subsequently used to calculate the burst pressure (P) according to the equation below (**Equation 1**).

$$Hr = \frac{P}{Q} \tag{1}$$

To isolate the contribution of the device itself, the hydraulic resistance associated with the instrumental setup was first measured in the absence of the microfluidic chip ($Hr_{system}$). The total hydraulic resistance measured with the chip connected ($Hr_{system+device}$) was then subtracted from $Hr_{system}$, yielding the hydraulic resistance exclusively attributable to the device.

Experimental burst-pressure measurements were performed utilizing a PDMS microchannel with reduced dimensions (140 µm width, 45 µm height, and 25 mm length; **Figure S1**). The channels were fabricated by replica molding using a photolithographically patterned master mold, as previously reported.[1] Owing to the increased hydraulic resistance associated with this geometry, the setup exhibited sufficient sensitivity to resolve the pressure drop arising from the PDMS/PDMS device (reversibly bonded, as described in main text). After correction by the $Hr_{system}$ value, the hydraulic resistance of the microfluidic channel was determined to be 0.4 kPa min µL$^{-1}$. This manually assembled device remained leakage-free up to 150 µL min$^{-1}$ (see **Figure S1**), which corresponds to a burst pressure of 63 kPa.[2] These values are consistent with those typically reported for reversible PDMS bonding approaches.

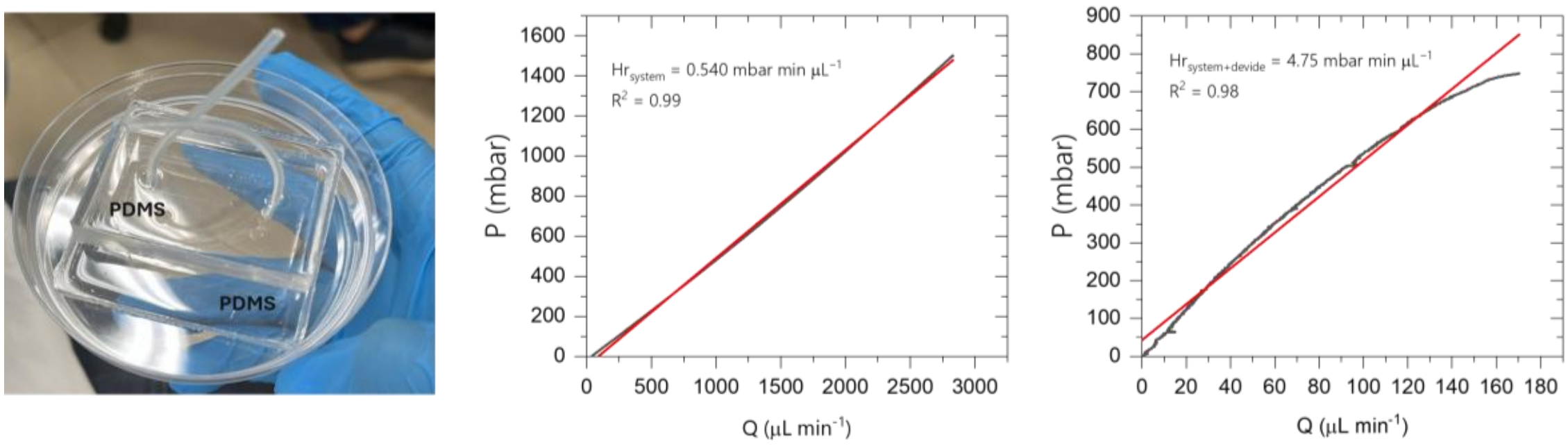


**Figure S1. Burst pressure measurements.** Microfluidic device with reduced channel dimensions (on the left) and the corresponding pressure ramps measured for the pumping system alone (in the middle) and after connection to the device (on the right).

## 2. Curved-well geometry

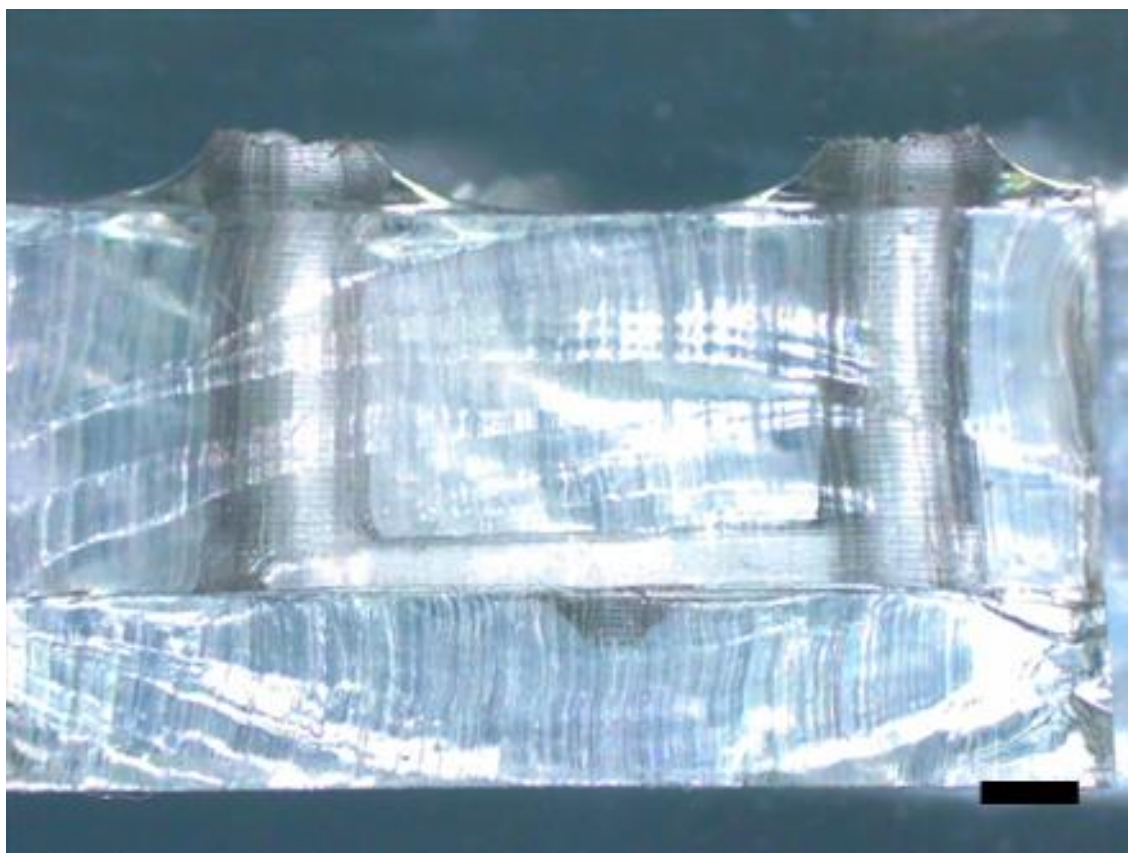

**Figure S2.** Chip with a curved-well design to evaluate the reduction in bubble formation. Scale bar: 1 mm.

## 3. Surface Passivation (Pluronic F127)

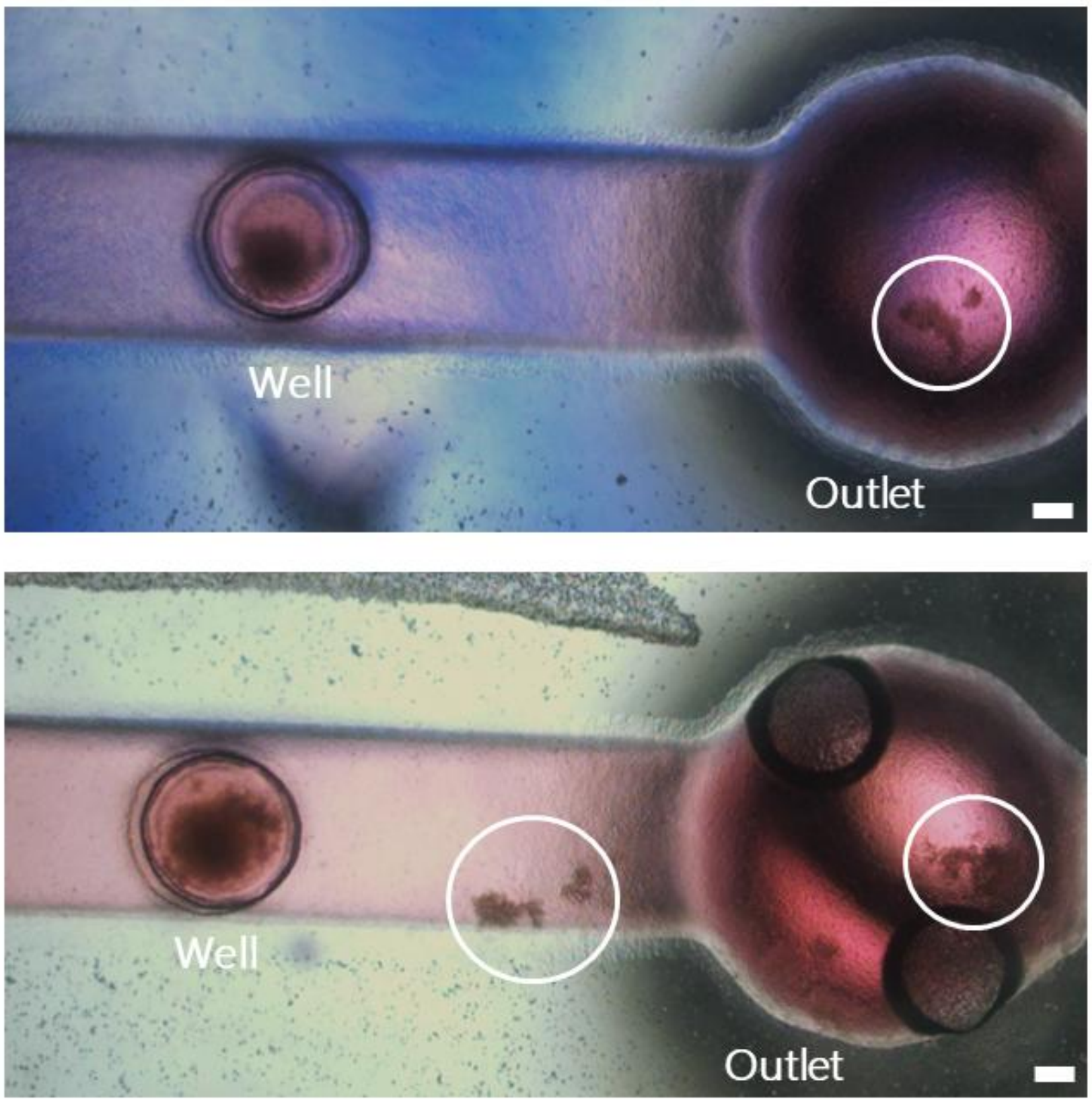


**Figure S3.** Cell adhesion of MDA-MB-231 cells at the microchannel inlets after 5 h incubation with 1% (w/w) Pluronic F127 at 37 °C. Scale bar: 200 μm.

## 4. Spheroid Formation Using Different Cell Seeding Densities

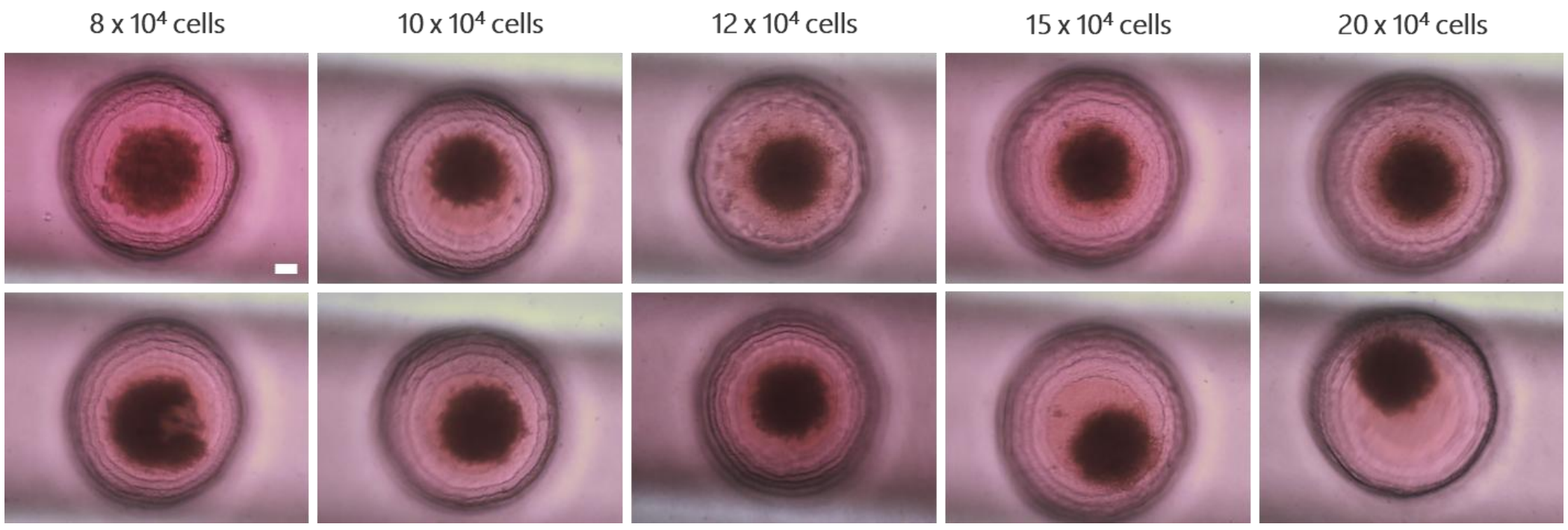


**Figure S4.** MDA-MB-231 spheroids formed in the reversible microfluidic device at different cell seeding densities. Scale bar: 100 μm.

## 5. Recovery of spheroids from the microfluidic channel after sequential collection steps

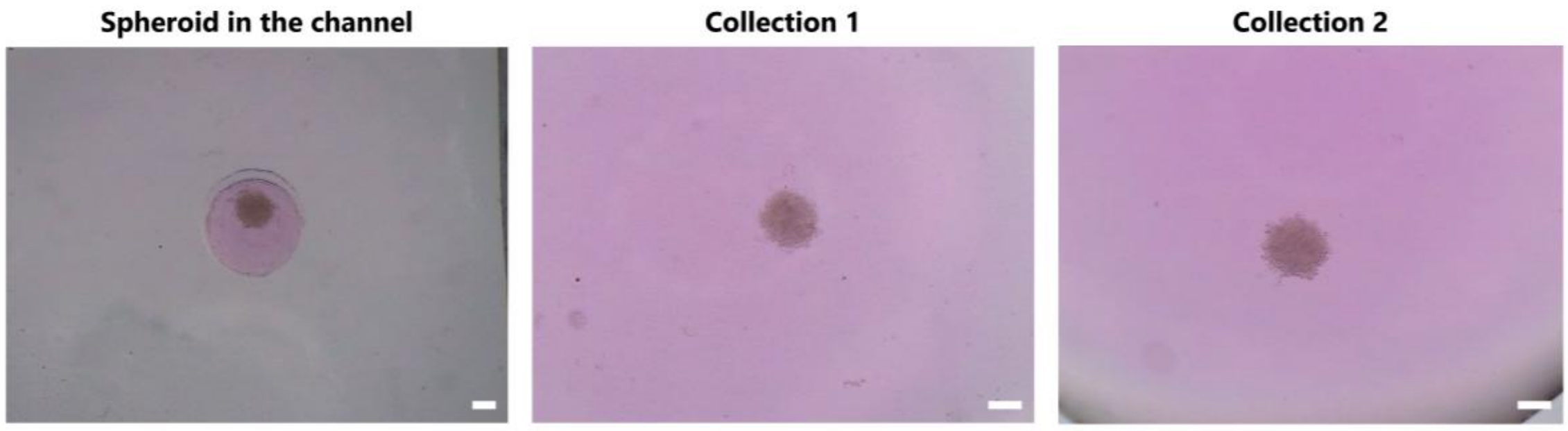


**Figure S5.** Representative bright-field micrographs showing the spheroid inside the microfluidic channel (“Spheroid in the channel”) and after two sequential recovery steps (“Collection 1” and “Collection 2”). The images demonstrate the successful retrieval and preservation of spheroid integrity following collection from the microfluidic platform. Scale bars: 200 μm.

## 6. Implementation of dynamic perfusion in the microfluidic platform using a peristaltic pump

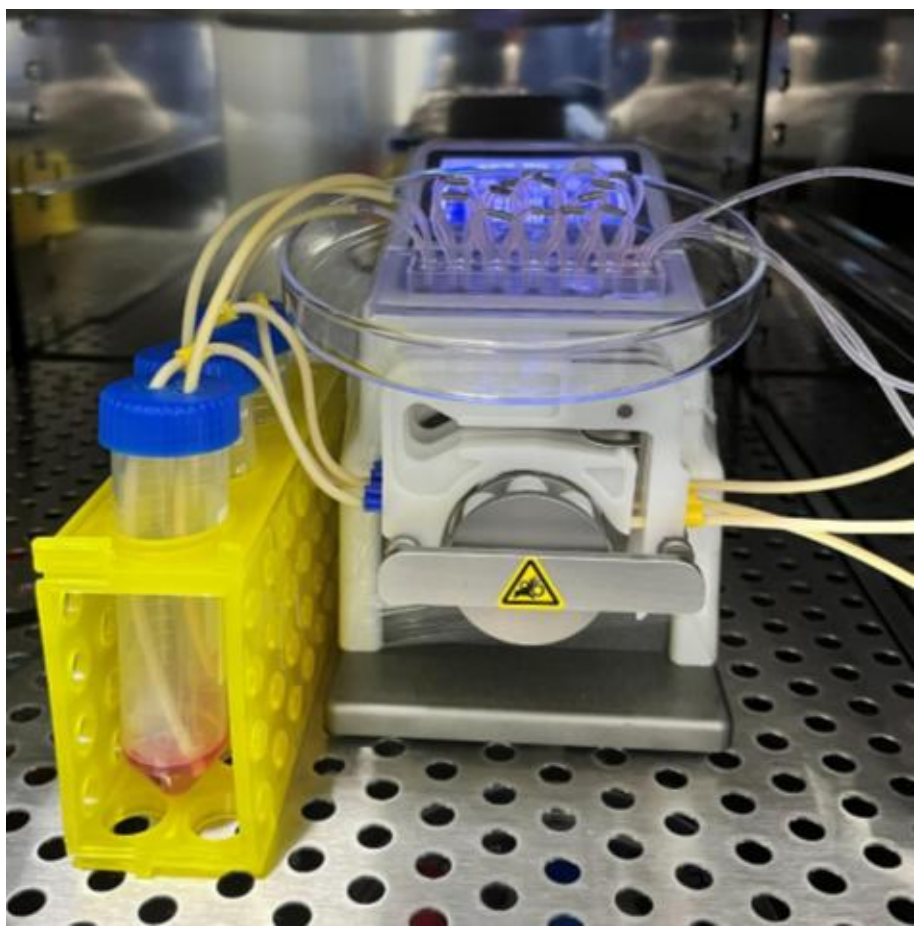

**Figure S6.** Photograph of the microfluidic perfusion setup using a peristaltic pump–based recirculation system.

## 7. Videos

**Video S1.** Introduction of the aqueous medium resulted in bubble formation, preventing complete microwell filling.

**Video S2.** Ethanol pre-treatment effectively mitigated bubble formation within the microwells under manual pipetting.

**Video S3.** Dye imaging confirmed progressive fluid exchange, with complete replacement achieved after five cycles.

## References


(1) da Silva, P. H. N.; Corsato, P. C. R.; Silva, C. O.; Pimentel, G. J. C.; Hryniewicz, B. M.; Bragantin, B.; Costa, R. S.; Shimizu, F. M.; Sousa Ribeiro, I. R. S.; Lima, R. S. Touch-Enabled Reversible Microfluidic Ultradense Chips for Convenient, High-Throughput Electrochemical Assays. *ACS Appl. Mater. Interfaces* **2025**, *17*, 45847-45858.

(2) da Silva, P. H. N.; Corsato, P. C. R.; Silva, C. O.; Pimentel, G. J. C.; Hryniewicz, B. M.; Bragantin, B.; Costa, R. S.; Shimizu, F. M.; Sousa Ribeiro, I. R. S.; Lima, R. S. Correction to "Touch-Enabled Reversible Microfluidic Ultradense Chips for Convenient, High-Throughput Electrochemical Assays". *ACS Appl. Mater. Interfaces* **2025**, *17*, 71063-71065.
